# Budding Yeast *SLX4* Contributes to the Appropriate Distribution of Crossovers and Meiotic Double-Strand Break Formation on Bivalents During Meiosis

**DOI:** 10.1534/g3.116.029488

**Published:** 2016-05-06

**Authors:** Mika Higashide, Miki Shinohara

**Affiliations:** * Department of Integrated Protein Functions, Institute for Protein Research, Osaka University, 565-0871, Japan; † Department of Biological Science, Graduate School of Science, Osaka University, 565-0871, Japan

**Keywords:** meiotic recombination, crossover control, chromosome segregation, endonuclease

## Abstract

The number and distribution of meiosis crossover (CO) events on each bivalent are strictly controlled by multiple mechanisms to assure proper chromosome segregation during the first meiotic division. In *Saccharomyces cerevisiae*, Slx4 is a multi-functional scaffold protein for structure-selective endonucleases, such as Slx1 and Rad1 (which are involved in DNA damage repair), and is also a negative regulator of the Rad9-dependent signaling pathway with Rtt107. Slx4 has been believed to play only a minor role in meiotic recombination. Here, we report that Slx4 is involved in proper intrachromosomal distribution of meiotic CO formation, especially in regions near centromeres. We observed an increase in uncontrolled CO formation only in a region near the centromere in the *slx4*∆ mutant. Interestingly, this phenomenon was not observed in the *slx1*∆, *rad1*∆, or *rtt107*∆ mutants. In addition, we observed a reduced number of DNA double-strand breaks (DSBs) and altered meiotic DSB distribution on chromosomes in the *slx4*∆ mutant. This suggests that the multi-functional Slx4 is required for proper CO formation and meiotic DSB formation.

Meiotic crossover (CO) formation is essential for proper segregation of homologous chromosomes during meiosis I, and the number of COs per bivalent is strictly regulated. CO formation originates from Spo11-dependent meiotic double-strand break (DSB) formation at recombination hot spots (Keeney 2001). A hot spot is generally defined not by specific DNA sequences, but by an open chromatin structure and epigenetic marks of histone modification (Borde *et al.* 2009; Buard *et al.* 2009; Lichten and de Massy 2011; Bani Ismail *et al.* 2014). In addition, whole-genome mapping of meiotic DSBs indicates that the distribution of meiotic DSBs is not uniform, and there are regions with few DSBs (cold spot), including those near centromeres and telomeres (Buhler *et al.* 2007).

Meiotic DSBs are repaired by Rad51- and Dmc1-mediated homologous recombination (Bishop 1994; Shinohara *et al.* 1997). Zip-Mer-Msh (ZMM) components, especially Zip3, Zip1, Msh4, and Msh5, are essential for both CO formation and its control, such as CO assurance and CO interference (Hollingsworth *et al.* 1995; Agarwal and Roeder 2000; Snowden *et al.* 2004; Shinohara *et al.* 2008; Nishant *et al.* 2010). In contrast, other ZMM components, especially Spo22, Zip2, and Spo16, are involved in the elongation of transverse element, which consists of Zip1 polymerization, during synaptonemal complex (SC) formation (Chua and Roeder 1998; Tsubouchi *et al.* 2006; Shinohara *et al.* 2008).

Slx4 functions as a scaffold for various structure-selective endonucleases that are involved in repairing many kinds of DNA lesions. Slx4 forms a complex with Rad1-Rad10 (ERCC1-XPF in mammals), with Slx1 in budding yeast and mammals, and with Mus81-Eme1, an ortholog of yeast Mms4, in mammals (Munoz *et al.* 2009; Rouse 2009). Rad1-Rad10 is a 3′-flap end nuclease, and is involved in nucleotide excision repair and recombination (Schiestl and Prakash 1990; Mazon *et al.* 2012; Munoz-Galvan *et al.* 2012; Saito *et al.* 2012). Slx1-Slx4 cleaves the 5′-flap as well as the replication fork structure *in vitro* (Fricke and Brill 2003), and plays a minor role as a resolvase of Holliday junctions during meiosis (De Muyt *et al.* 2012; Zakharyevich *et al.* 2012). In addition, Slx4 is involved in interstrand cross-link (ICL) repair, and is also known as FANC-P, which is responsible for one subgroup of Fanconi anemia in humans (Kim *et al.* 2011; Stoepker *et al.* 2011). In addition to its interactions with nucleases, Slx4 also interacts with the DNA damage response (DDR) component Rtt107. The Rtt107-Slx4 complex is involved in suppression of the Dpb11-Rad9-related signaling pathway (Ohouo *et al.* 2010, 2013).

In yeast meiotic CO formation, Mlh1-Mlh3 functions as a main player in pro-CO intermediate joint molecule resolution (Zakharyevich *et al.* 2012). Thus, Slx1-Slx4 has a redundant role with Mus81-Mms4 and Yen1, as well as a very minor role in meiotic recombination (De Muyt *et al.* 2012; Zakharyevich *et al.* 2012). In contrast, in *Caenorhabditis elegans*, SLX-1–HIM-18/SLX4 is involved in suppression of CO formation at the center region of the chromosomes through a function of the plant homeodomain (PHD) finger in the SLX-1 protein (Saito and Colaiacovo 2014).

## Materials and Methods

### Yeast strains

All genotypes of *Saccharomyces cerevisiae* strains used in this study are shown in Supplemental Material, Table S1. Deletion alleles of *SLX4*, *SLX1*, *RTT107*, and *RAD1* were constructed using PCR-mediated gene disruption (Wach *et al.* 1994). The *cup2-B* and *ade6-B* mutations for MSY4304 were introduced by insertion of a *Bam*HI linker at the first ATG site of each gene by using site-direct mutagenesis. The *met13-B* and *trp5-S* mutations were introduced by crossing with strain NHY942 (a gift from Dr. Neil Hunter) which is *MAT*a parent of NHY957 (de los Santos *et al.* 2003). The original *SPO11-3FLAG* and *spo11-Y135F* strains were gifts from Dr. K. Ohta and Dr. S. Keeney, respectively (Diaz *et al.* 2002; Sasanuma *et al.* 2007).

### Yeast meiosis time course analysis

*S. cerevisiae* strains derived from SK1 background NKY1551 (Storlazzi *et al.* 1996) were used for meiotic cytological analysis, and western blot and Southern blot analyses. Meiotic time course experiments were carried out as described (Shinohara *et al.* 2003).

### Cytological analysis

Cytological analysis by immunostaining of yeast meiotic nuclear spreads was performed as described (Shinohara *et al.* 2008). Stained samples were observed using an epifluorescence microscope (Axioskop2, Zeiss), with LED fluorescence light sources (X-Cite; Excelitas Technologies), and a 100 × objective (Axioplan, NA1.4, Zeiss). Images were captured with a CCD camera (Retiga, Qimaging), and processed using iVision (BioVision Technologies) and Photoshop (Adobe) software. More than 100 nuclei were counted for each sample, and more than five foci-positive nucleus in a cell indicated a focus-positive cell. Antibodies used for this study were anti-Zip1 [rat, 1:500 (Shinohara *et al.* 2008)], anti-Rad51 [rabbit, 1:500 (Shinohara *et al.* 2015) or guinea pig, 1:500 (Shinohara *et al.* 2000)], and anti-Dmc1 [rabbit, 1:500 (Hayase *et al.* 2004)].

### Western blotting

Whole-cell lysates of meiotic cells were extracted with the TCA precipitation method (Sasanuma *et al.* 2013), and then proteins were separated on SDS-PAGE gels and transferred to PVDF membranes (Immobilon-FL, Millipore). The following antibodies were used for western blotting: anti-DYKDDDDK tag (1E6, Wako), anti-Hop1 ((Iwasaki et al. 2016), guinea pig, 1:1000), anti-Hop1-pT318 ((Iwasaki et al. 2016), rabbit, 1:1000), and anti-tubulin (MCA77G, AbD Serotec). Primary antibodies were visualized with Alexa Fluor 680-conjugated (Molecular Probes) or IRDye 800-conjugated (LI-COR Biosciences) secondary antibodies using an Odyssey infrared imaging system (LI-COR Biosciences). The density of each signal was determined by using ImageStudio v3.1 software (LI-COR Biosciences).

### Yeast genetic analysis of meiotic recombination

For tetrad analysis, zygotes were generated by 3-hr matings of each parental haploid strain derived from MSY4304 or MSY4245 (Table S1), and then transferred to a sporulation medium plate (0.3% potassium acetate, 0.02% raffinose, and 2% agar) and incubated for 48 hr at 30°. Genetic distances between markers and CO interference were analyzed by using the MacTetrad 6.9.1 program (ftp://130.14.250.7/repository/yeast/mactetrad/) as described (Shinohara *et al.* 2008). Map distances were determined using Perkins equation, and SEMs were calculated using the Stahl Lab online tool (http://www.molbio.uoregon.edu/~fstahl). At least four independent crosses were analyzed for each strain.

### Southern blotting

Southern blotting was carried out as described (Storlazzi *et al.* 1996; Shima *et al.* 2005; Shinohara and Shinohara 2013). Genomic DNA from NKY1551-derived yeast strains was digested using *Pst*I for DSB detection; *Xho*I for inter-homolog CO recombination (IHR) detection: and *Mlu*I, *Xho*I and *Bam*HI for hetero-duplex (HD) detection. DNAs were transferred onto nylon membranes (ClearTrans, Wako) by capillary transfer. Probes for Southern blotting were prepared using pNKY291 for DSB and pNKY155 for CO/NCO (non-CO) detection (Xu *et al.* 1995). Detection of DSBs at the *ELO2* locus was carried out as described (Gothwal *et al.* 2016). Probes were labeled with α-[^32^P]-dATP using random labeling with the Klenow fragment (3′-5′ -exo) (NEB) and random dN6 (NEB). Blots were detected using a Phosphorimager BAS5000 (Fuji film) and quantified using ImageQuant software (GE Healthcare).

### Contour-clamped homogeneous electrical field (CHEF) analysis

Pulsed-field gel electrophoresis (PFGE) to detect the whole-chromosome distribution of meiotic DSBs was performed as described (Bani Ismail *et al.* 2014). Genomic DNA from NKY1551-derivatived yeast strains was prepared in agarose plugs and run under the following conditions: 120° angle, 6 V/cm, and 48 hr with the CHEF DR-III (Bio-Rad), with 25 sec to 125 sec as the switch time. Signals were visualized by Southern blotting as described above. Probes for Southern blotting were prepared using *CHA1* for chromosome III and *CUP2* for chromosome VII (Bani Ismail *et al.* 2014).

### Spo11-bound oligo DNA detection

The DNA fragment covalently bound to Spo11 protein was isolated as described (Pan and Keeney 2009). Briefly, Spo11-3FLAG was immunoprecipitated with anti-DYKDDDDK (1E10, Wako) and Dynabeads Protein G (Veritas) from TCA-treated whole meiotic cell extract. DNA fragments in the immunoprecipitates were labeled with α- [^32^P]-dCTP (NEG531Z, Perkin Elmer) by using terminal transferase (NEB). ^32^P signals were detected with a Phosphorimager BAS5000 after separation by SDS-PAGE. Spo11-3FLAG protein in the immunoprecipitates was detected by western blotting with TrueBlot HRP-conjugated anti-Mouse Ig (Rockland), and then signals were visualized with the ImageQuant LAS4000 (GE healthcare) after treatment with ECL Prime Western blotting detection reagent (GE Healthcare).

### Data availability

The authors state that all data necessary for confirming the conclusions presented in the article are represented fully within the article.

## Results

### slx4∆ cells are delayed in meiosis progression in a meiotic DSB-dependent manner

**Figure 1:**
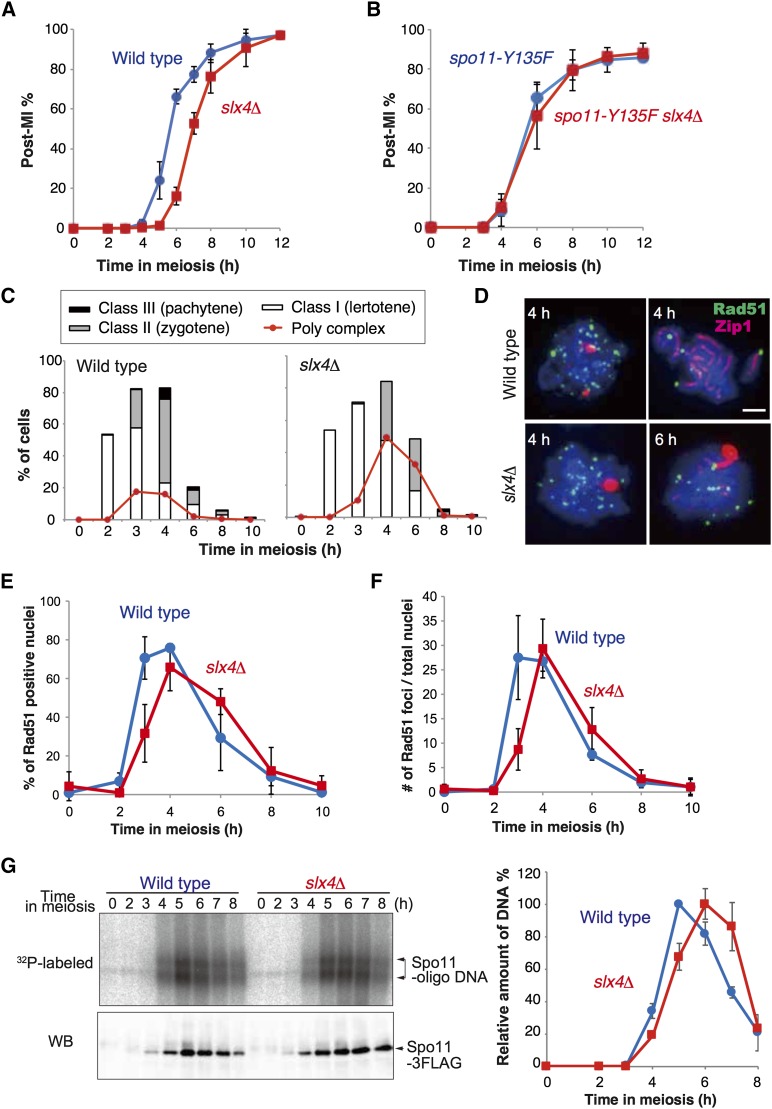
*slx4*∆ cells are delayed in meiosis progression with a defect in Zip1 elongation and Rad51 assembly. (A) Meiosis progression was analyzed in wild type (NKY1551) and *slx4*∆ (MHY24) strains. The percentage of cells containing two, three, and four nuclei per ascus (post-MI %) is shown in the graph. Error bars indicate the SD from at least three independent trials. (B) Meiosis progression was analyzed in *spo11-Y135F* (MSY3699) and *spo11-Y135F slx4*∆ (MHY365) strains. Error bars indicate the SD from at least three independent trials. (C) SC elongation was analyzed in wild type and *slx4*∆ by immunostaining for Zip1 at each time point. A representative graph showing percentages for each class—class I (leptotene), class II (zygotene), and class III (pachytene)—for SC elongation for each time point is shown. The percentages of cells with poly complex structures of Zip1 staining are shown in red. (D) Representative images of meiotic nuclear spreads from each indicated time point that were costained with anti-Rad51 (green) and anti-Zip1 (red) in wild type and *slx4*∆. Scale bar indicates 2 µm. (E) Percentages of Rad51-positive nuclei in wild type and *slx4*∆ at each time point during meiosis. Error bars indicate the SD from at least three independent trials. (F) Average numbers of Rad51 foci per nucleus at each time point were analyzed in wild type and *slx4*∆. At least 100 nuclei were analyzed for each time point. Error bars show the SEM from at least three independent trials. (G) Representative ^32^P-labeled DNA fragments covalently bound to Spo11-3FLAG (upper) and Spo11-3FLAG protein (lower) in immunoprecipitates from *SPO11-3FLAG* (wild type, MSY5089) and *slx*4∆ *SPO11-3FLAG* (*slx*4∆, MHY471) are shown. Average of relative DNA fragment signals at each time point, which was shown as percent of peak amount of signal in wild type (5 hr), were shown in graph. Error bar shows SD from three independent trials.

Although Slx4 is involved in a minor pathway to resolve Holliday junctions with Slx1, in contrast to the *slx1*∆ mutant, the *slx4*∆ mutant has delayed meiosis progression (De Muyt *et al.* 2012; Zakharyevich *et al.* 2012). We confirmed that the *slx4*∆ mutant showed a 1.3-hr delay in meiosis I entry (Figure 1A). We then analyzed a *spo11* catalytic mutation, *spo11-Y135F*, which suppressed the delayed meiosis in the *slx4*∆ mutant (Figure 1B). This suggested that the delay in *slx4*∆ is caused by a post-DSB event.

To confirm the cause of the delay, we analyzed Zip1 elongation. Zip1 is a component of the central element of the SC, which is visible as dotty (class I; leptotene), partially elongated (class II; zygotene), and fully elongated (class III; pachytene) structures according to the progression of prophase I in wild type (Figure 1C). In the *slx4*∆ mutant, whereas the timing of appearance of class I Zip1 was normal, the appearance of the partially elongated Zip1 signal, and also the disappearance of the Zip1 signal that occurs soon after 6 hr in the wild type, was delayed in the *slx4*∆ mutant (Figure 1C and Figure S1A). In addition, elongation of Zip1 was affected as compared with wild type, and also the poly complex structure, which is a marker of an SC elongation defect (Shinohara *et al.* 2008), was increased in the *slx4*∆ mutant (Figure 1, C and D). This suggested that meiosis progression before leptotene would be normal in the *slx4*∆ mutant.

Although the initial timing of Zip1 assembly was normal, we observed a 0.8-hr delay in the appearance of Rad51 foci in the *slx4*∆ mutant (Figure 1E and Figure S1B). In addition, disappearance of Rad51 foci was also delayed (0.7 hr) in the *slx4*∆ mutant. There was, however, no significant difference in the average numbers of Rad51 foci per nucleus between wild-type and *slx4*∆ cells at each meiotic chromatin at their peak abundance, after 4 hr in meiosis (Figure 1F). In addition, there was no difference in the life span of Rad51 focus positive nuclei in the *slx4*∆ mutant compared with that in wild type (3.04 ± 0.64 hr and 2.95 ± 0.57 hr, respectively, Figure S1B), thus this suggests that turnover of Rad51 foci was not affected in the *slx4*∆. These observations suggest that Slx4 plays a role in the leptotene-to-zygotene transition, perhaps specifically in the formation of DSBs, and their extensive resection to promote Rad51 assembly. Then, we observed reduced amount of Spo11-bound oligomeric DNA fragment (Spo11-oligo) in the early phase (at 3–5 hr) in the *slx4*∆ mutant (Figure 1G), similar to the Rad51 focus number (Figure 1F). In contrast, the amount of Spo11-oligo at the peak point (6 hr) was distinguishable from that in wild type. This strongly suggests that Slx4 is required for efficient DSB formation.

### Delay of meiosis progression in the slx4∆ mutant occurs independently of the Slx4-related components

**Figure 2:**
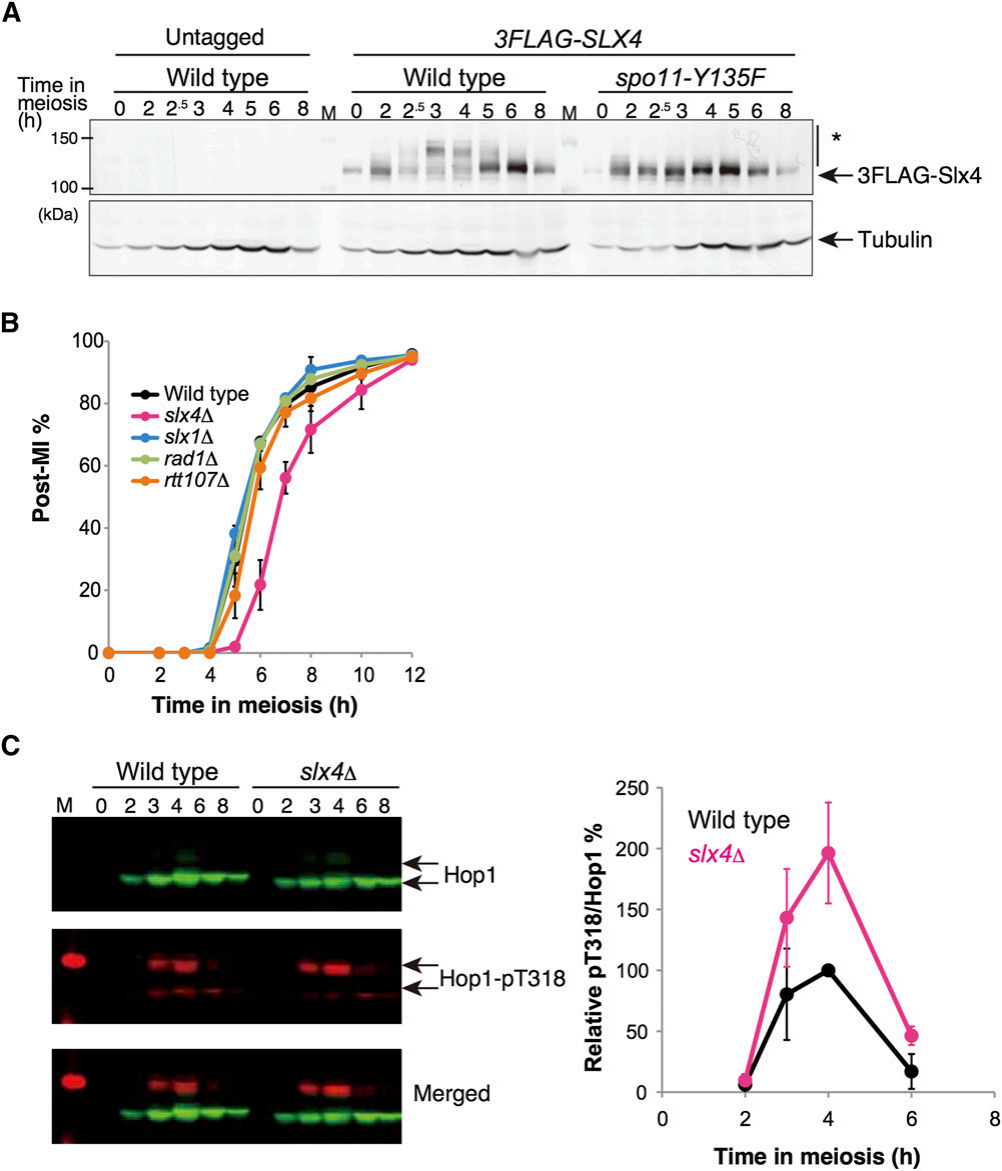
The multiple functions of Slx4 are required for proper meiosis. (A) Phosphorylation of 3FLAG-Slx4 was analyzed during meiosis. Whole-cell extracts from wild type (untagged; NKY1551), *3FLAG-SLX4* (MHY129), and *spo11-Y135F 3FLAG-SLX4* (MHY187) meiotic cells were analyzed by western blotting with anti-FLAG and anti-tubulin antibodies. The asterisk indicates the phosphorylated signal. (B) Meiosis progression in wild type (NKY1551), *slx4*∆ (MHY24), *slx1*∆ (MHY68), *rad1*∆ (MHY96), and *rtt107*∆ (MHY235) was analyzed as described in Figure 1A. (C) Phosphorylation of Hop1 at T318 in wild type (NKY1551) and *slx4*∆ (MHY24) was analyzed by western blotting with anti-Hop1 (green) and anti-Hop1-pT318 (red) (left). The relative ratio of pT318 signal to the Hop1 signal is shown in the graph (right). Error bars show SD from more than three independent trials.

Slx4 is phosphorylated by the Mec1 and Tel1 kinases after DNA damage (Flott *et al.* 2007; Toh *et al.* 2010). We analyzed Slx4 phosphorylation during meiosis. We conjugated Slx4 protein with 3 × FLAG epitope at the N -terminus (3FLAG-Slx4), and confirmed that the tagging does not affect meiosis progression (Figure S2A). We analyzed Slx4 protein during meiosis by western blotting with an antibody against FLAG. We detected an increase in 3FLAG-Slx4 expression during meiosis, and also multiple slower-migrating signals at 2.5 to 4 hr after meiosis entry, such that most of the 3FLAG-Slx4 protein was hyper-shifted at 3 to 4 hr (Figure 2A) when the appearance of Rad51 foci peaks (Figure 1E). The hyper-shifted 3FLAG-Slx4 was undetectable in *spo11-Y135F* mutant (Figure 2A). In addition, we confirmed that mobility of the hyper-shifted signal was indistinguishable from the signals induced by DNA damage in both mitotic and meiotic cells (Figure S2B). This indicated that Slx4 is phosphorylated as a result of not only accidental DSBs induced during vegetative growth but also programmed DSBs induced by Spo11 during meiosis.

Tel1/Mec1-dependent phosphorylation of Slx4 is related to the Rad1-Rad10 endonuclease activity of cleaving nonhomologous tails in the single-strand annealing (SSA) pathway (Toh *et al.* 2010). We thus examined the contribution of Rad1-Rad10 nuclease in meiosis progression. In contrast to the *slx4*∆ mutant, we did not observe any delay in meiosis in the *rad1*∆ mutant (Figure 2B). We also analyzed mutations in two additional Slx4-related components: Slx1 and Rtt107. Rtt107 is involved in the regulation of Rad53 activity through a mechanism referred to as dampens checkpoint adaptor-mediated phosphor-signaling (DAMP) during mitosis (Ohouo *et al.* 2010). However, we did not observe any delay in meiosis in these mutants (Figure 2B). On the other hand, it is known that *mms4* and *mus81* mutants show a delay in meiosis progression, and, then, the *slx4*∆ *mms4* meiotic null double mutant shows additive delays (de los Santos *et al.* 2003; De Muyt *et al.* 2012).

We next analyzed Hop1 expression and phosphorylation to determine whether Slx4 is required in the meiotic DSB-related checkpoint pathway. Hop1, a multi-functional protein, is a meiosis-specific component of the axial structure of the SC, and Mec1/Tel1-dependent phosphorylation of Hop1 is essential for its function (Carballo *et al.* 2008). We used an antibody against whole Hop1 protein, and an antibody specific for phospho-T318 of Hop1 to monitor Mec1/Tel1-dependent Hop1 phosphorylation. Expression of Hop1 was detected from 2 hr after meiosis entry in both wild type and the *slx4*∆ mutant (Figure 2C). This also indicates that meiosis progression before leptotene would be normal in the *slx4*∆ mutant. Phosphorylation of Hop1 began to appear after 3 hr in meiosis, with robust phosphorylation detected at 4 hr in wild type and the *slx4*∆ mutant. Interestingly, quantification of the signals indicated that Hop1 phosphorylation level was increased in the *slx4*∆ mutant cells (Figure 2C).

### slx4∆ cells have altered intrachromosomal distribution of COs on chromosomes III and VII

**Figure 3:**
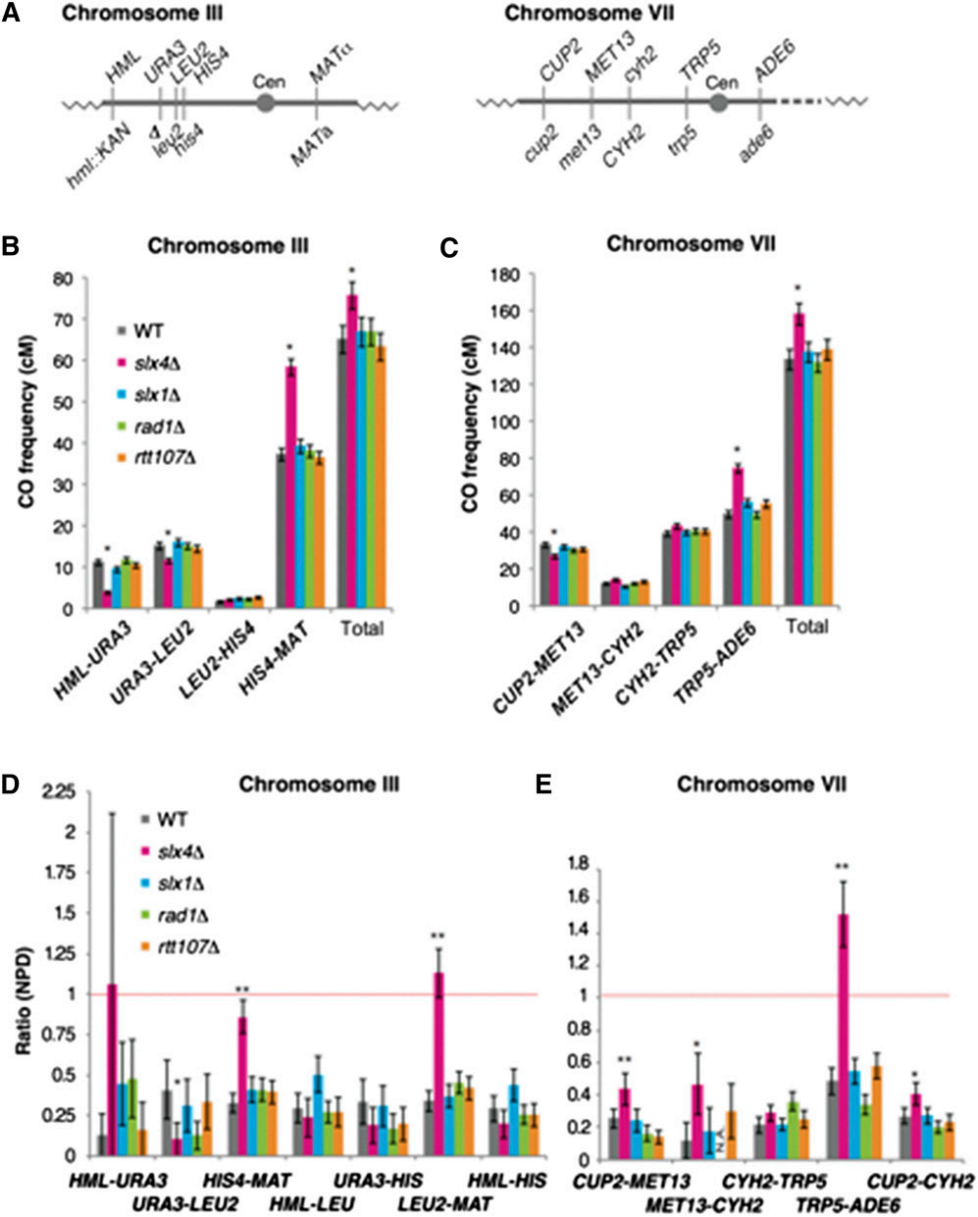
CO distribution and CO interference are affected in *slx4*∆ cells in the chromosomal region that includes the centromere. (A) Schematics show physical maps of genetic markers used for the genetic analysis and centromere (Cen) on chromosomes III or VII. (B) Genetic CO frequencies for each interval on chromosome III in wild type (NKY4304/4245), *slx4*∆ (MSY4930/4910), *slx1*∆ (MSY5314/5282), *rad1*∆ (MSY5624/5622), and *rtt107*∆ (MSY5622/5159) are shown. (C) Genetic CO frequencies for each interval on chromosome VII are shown. (D) NPD ratios on chromosome III were analyzed using Papazian’s method in wild type (NKY4304/4245, *n* = 1341), *slx4*∆ (MSY4930/4910, *n* = 1682), *slx1*∆ (MSY5314/5282, *n* = 1362), *rad1*∆ (MSY5624/5622, *n* = 1724), and *rtt107*∆ (MSY5622/5159, *n* = 1192). Error bars show the SEM. Details are shown in Table S3. (E) NPD ratios on chromosome VII are shown. Error bars in (B) to (E) indicate the SEM, and asterisks indicate a significant difference between the values in wild type based on Perkins formula (** *P* < 0.01, * *P* < 0.05). All values were calculated using the Stahl Laboratory on-line tool.

A previous genetic analysis in budding yeast revealed that *slx4*∆ mutant cells show as significant increase in CO frequency in the *HIS4LEU2-MAT* interval, but not in the interval *URA3-HIS4LEU2* in the strain that includes the *HIS4-LEU2* hot spot on chromosome III (Zakharyevich *et al.* 2012). We reanalyzed the CO frequency in additional intervals including the *HIS4-MAT* interval on chromosome III and also several intervals on chromosome VII (Figure 3A) to compare COs in different chromosomes of different length. We compared the genetic length (in centimorgans) of each interval among wild type, and the *slx4*∆, *slx1*∆, *rtt107*∆, and *rad1*∆ mutants. First, we confirmed that *slx4*∆ does not show any defect in spore viability (Mullen *et al.* 2001); in addition, the *SLX4*-related mutants *slx1*∆, *rad1*∆, *rad10*∆, and *rtt107*∆ showed no changes in spore viability compared with wild type (Table S2).

We confirmed that the CO frequency on chromosome III within the *HIS4-MAT* interval, which includes the centromere, was significantly increased in *slx4*∆ (58 ± 2.0 cM) as compared with wild type (37 ± 1.5 cM) (Figure 3B and Table S3). In contrast, CO frequency was significantly reduced in the *HML-URA3* interval, and slightly decreased in the *URA3-LEU2* interval. Changes in CO frequencies were not observed in other *SLX4*-related mutants, meaning CO frequencies in the *HIS4-MAT*, *URA3-LEU2*, and *HML-URA3* intervals in *slx1*∆, *rtt107*∆, and *rad1*∆ mutants were indistinguishable from those of wild type. When the CO frequencies were summed, the *slx4*∆ mutant showed an increase in CO frequency in the *HML*-though-*MAT* region on chromosome III (Figure 3B).

Similar increases and decreases in CO frequencies were observed on chromosome VII, one of the largest chromosomes in budding yeast. In the *slx4*∆ mutant, CO frequency was significantly increased in the *TRP5-ADE6* interval, which includes the centromere. It was unchanged in the *MET13-CYH2* and *CYH2-TRP5* intervals, but was significantly decreased in the *CUP2-MET13* interval. However, these tendencies were not observed in the other *SLX4*-related mutants (Figure 3C and Table S3). Again, the total CO frequency across these intervals on chromosome VII was increased in *slx4*∆. In contrast, there were no significant differences in the noncrossover (NCO) frequencies, observed as non-Mendelian segregation, at each genetic locus in *slx4*∆ and in the *SLX4*-related mutants, and also that in wild type (Table S4). These results suggested that *SLX4* is involved in the regulation of CO distribution along each chromosome.

To determine the function of Slx4 and Slx4-related components in CO control, we analyzed CO interference for each interval on both chromosome III and chromosome VII by an analysis with Papazian’s equation (Figure 3, D and E, and Table S3), as well as the coefficient of coincidence (COC) (Table S5). In wild type, the ratios of the observed to expected number of nonparental di-types (NPDs) were < 0.5 for all intervals (Figure 3, D and E), indicating the presence of CO interference. In the *slx4*∆ mutant, we observed abolished CO interference in the *HIS4-MAT* and *LEU2-MAT* intervals (0.86 ± 0.1 and 1.13 ± 0.15, respectively) on chromosome III and in the *TRP5-ADE6* interval (1.52 ± 0.2) on chromosome VII (Figure 3, D and E, and Table S3). Interestingly, all three intervals include the centromere. Although the ratio of the observed number of NPDs to the expected number of NPDs was almost the same for the *HML-URA3* interval, it was not significant because of a small number of NPD tetrads in *slx4*∆ (Table S3). In addition, we observed compromised CO interference in the *CUP2-MET13* and *MET13-CYH2* intervals in *slx4*∆ as compared with that in wild type (Figure 3E and Table S3). In contrast, there was a significantly greater amount of CO interference in the *URA3-LEU2* interval in *slx4*∆ than in wild type. In support of this finding, we observed weakened CO interference in the *HML-URA3-LEU2* region, for which the ratios of observed to expected consecutive COs were 0.491 (*P* < 0.001) and 0.724 (*P* = 0.41) in wild type and *slx4*∆, respectively, based on the COC method (Table S5).

### The slx4∆ mutant has a slight delay in meiotic DSB formation

**Figure 4:**
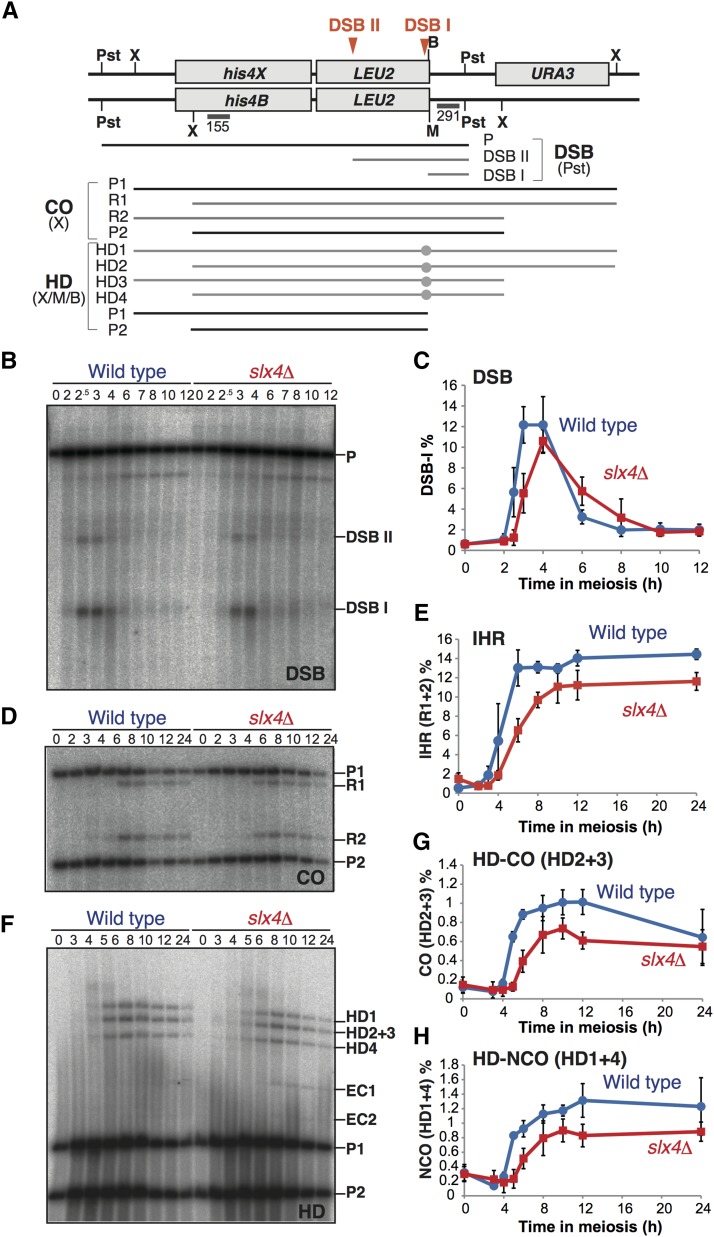
Physical analysis of meiotic recombination products at the *HIS4-LEU2* hot spot. (A) Schematic showing the *HIS4-LEU2* meiotic recombination hot spot. Diagnostic restriction enzyme sites are shown as X (*Xho*I), Pst (*Pst*I), M (*Mlu*I), and B (*Bam*HI). The sizes of meiotic DSBs (DSBs I and II) and parental band (P) detectable by probe 291, or of interhomolog COs (R1 and R2) and parental (P1 and P2) and heteroduplex (HD) intermediates (HD1–4) of homologous recombination and parental (P1 and P2) detectable by probe 155 are shown. (B) Representative Southern blotting of DSB detection in wild type (NKY1551) and *slx4*∆ (MHY24). Time in meiosis (in hours) is shown above the blot. (C) Quantification of DSB I signals in wild type and *slx4*∆ from Southern blotting as in (B). Error bars represent the SD (*n* = 4). (D) Representative Southern blotting of CO detection in wild type and *slx4*∆ from the same DNA samples as in (B). (E) Quantification of IHR signals (*i.e.*, R1 + R2) in wild type and *slx4*∆ from Southern blotting as in (D). Error bars represent SD (*n* = 3). (F) Representative Southern blotting of HD detection in wild type and *slx4*∆ from the same DNA samples as in (B). HD1 and HD4 correspond to NCO products, HD2 and HD3 correspond to the CO products as shown in (A). EC1 and EC2 are produced by ectopic recombination between *HIS4-LEU2* and *leu2*::*hisG* (Shinohara *et al.* 2003). (G) Quantification of HD-CO signals (*i.e.*, HD2 + HD3 signals, which correspond to CO intermediates) in wild type and *slx4*∆ from Southern blotting as in (F). Error bars represent the SD (*n* = 3). (H) Quantification of HD-NCO signals (*i.e.*, HD1 + HD4, which correspond to NCO intermediates) in wild type and *slx4*∆ from Southern blotting as in (F). Error bars represent the SD (*n* = 3).

To determine the cause of altered intrachromosomal CO distribution in the *slx*4∆ mutant, we analyzed the physical products of meiotic recombination at the *HIS4-LEU2* hot spot (Storlazzi *et al.* 1996) (Figure 4A). We observed a slight decrease in the genetic CO frequency in the overlapping intervals *URA3-LEU2* and *URA3-HIS4* (Figure 3B and Table S3). First, we analyzed programmed DSB formation as an initial event of meiotic CO formation by Southern blotting (Figure 4A and Figure S3). We observed a delay in the appearance and also the disappearance of meiotic DSBs in the *slx4*∆ mutant as compared with wild type (Figure 4, B and C), which corresponded temporally with the appearance and disappearance of Rad51 foci (Figure 1E). In addition, we observed a slight decreased in the peak amount of DSB formation in *slx4*∆ (10.6 ± 1.1% at 4 hr) as compared with that in wild type (12.2 ± 2.7% at 3 hr) (Figure 4C). This result suggested two possibilities: (i) the total amount of DSB formed at this hot spot was decreased, or (ii) DBSs were repaired more rapidly in the *slx4*∆ mutant. We observed the same amount of Spo11-oligo in *slx4*∆ at a peak point (5 hr) as compared with that in wild type (Figure 1G).

We analyzed interhomolog CO products (IHRs) at this locus (Figure 4A). We observed a delay in the appearance of the product, and a slight but significant reduction in the total amount of IHRs in the *slx*4∆ mutant (*P* = 0.006, at 6 hr) (Figure 4, D and E), although a previous study indicated that the *slx4*∆ single mutation has little effect on meiotic recombination (Zakharyevich *et al.* 2012). In addition, we analyzed both the CO products and NCO products separately with additional digests at the hetero-allelic restriction enzyme site *Mlu*I (Figure 4A). We observed a slight decrease in both CO and NCO products in the *slx*4∆ mutant as compared with wild type (Figure 4, F–H). This result corresponds to the result that the efficiency of DSB formation at the *HIS4-LEU2* hot spot was reduced in the *slx4*∆ mutant (Figure 4A). In addition, we observed an increase in extra bands, which are caused by ectopic recombination (Shinohara *et al.* 2003), in the *slx4*∆ mutant. An increase in ectopic recombination is also observed in the checkpoint mutants *mec1*, *rad24*, or *rad17*, and meiotic recombination, such as *dmc1* and *tid1/rdh54* (Grushcow *et al.* 1999; Shinohara and Shinohara 2013). These results suggest that the *slx4*∆ mutation compromises the strand-invasion process during meiotic recombination.

### Slx4 regulates the chromosome-wide distribution of meiotic DSB formation

**Figure 5:**
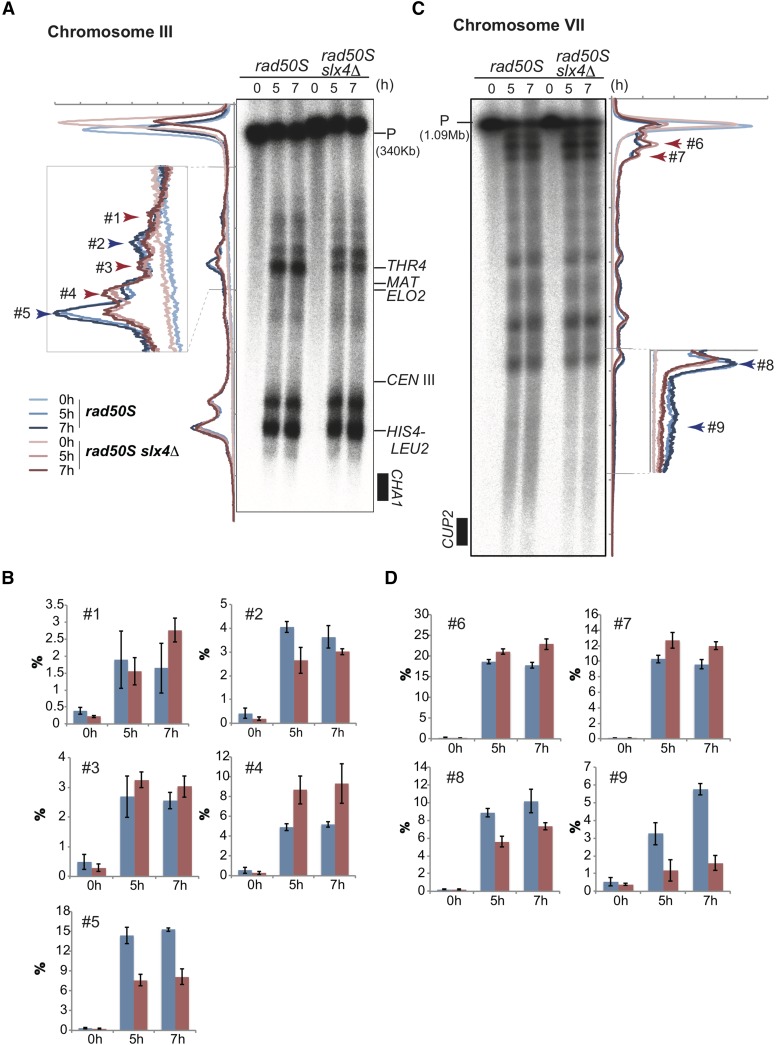
The distribution of meiotic DSBs is affected in *slx*4∆ on chromosomes III and VII. (A) Distribution of DSBs along chromosome III was analyzed by Southern blotting after separation by PFGE. (B) Average of quantified signals (#1–#5) shown in (A) at each time point in *rad50S* (blue) and *slx4*∆ *rad50S* (red). Error bars represent SD (*n* = 3). (C) Distribution of DSBs along chromosome VII was analyzed by Southern blotting after separation by PFGE. (D) Average of quantified signals (#6–#9) shown in (C) at each time point in *rad50S* (blue) and *slx4*∆ *rad50S* (red). Error bars represent SD (*n* = 3).

Because we observed altered genetic CO distribution for both chromosomes III and VII, and a reduction in the physical CO products corresponding to the reduced formation of DSBs at the *HIS4-LEU2* hot spot, we hypothesized that altered CO distribution may be caused by altered DSB formation in the *slx4*∆ mutant cells. To confirm this possibility, we analyzed chromosome-wide meiotic DSB distribution on chromosomes III (Figure 5, A and B) and VII (Figure 5, C and D) by using CHEF electrophoresis analysis. We used the *rad50S* background to make DSB signals more clear and to prevent DSB disappearance to facilitate quantification.

On chromosome III, we observed significant differences in DSB distribution (Figure 5A, #1–5, arrow marked). In some instances, the number of DSBs was reduced (#2 and #5, blue arrow) or elevated (#1, #3, and #4, red arrows) in *slx4*∆ as compared with wild type. Especially at sites #2, #4, and #5, the differences were notable (Figure 5B). In contrast, although we observed an increase in the genetic CO frequency in the *HIS4-MAT* interval, we did not observe a notable increase in DSB formation in this region with the CHEF analysis (Figure 5, A and B). We then analyzed site-specific DSB formation at the *ELO2* locus, which is known as a cold spot for DSB formation in wild type (Baudat and Nicolas 1997; Gothwal *et al.* 2016), and is located in the *HIS4-MAT* interval. We observed a slight increase in DSB formation in the *slx4*∆ mutant in the *rad50S* background at this locus (Figure S3). On chromosome VII, we observed a significant reduction in DSB formation in the region near the *CUP2* locus in the *slx4*∆ mutant (Figure 5, C and D; #9) as well as at site #8 (Figure 5, C and D). We originally considered region #9 to be a cold spot, as we did not observe any apparent DSB bands. We observed a significant reduction of genetic CO frequency in this region, *CUP2-MET13*, in *slx4*∆ (Figure 3C). In contrast, we observed a significant increase in the number of DSBs at sites #6 and #7 in *slx4*∆ (Figure 5, C and D). These results indicated that Slx4 is involved in DSB formation, and is required for the normal distribution of DSBs across each chromosome. In addition, we detected a reduced amount of Spo11-oligo DNA in the *slx4*∆ mutant compared with that in wild type in the early phase of meiotic recombination (Figure 1G).

## Discussion

SLX-1, together with SLX-4/HIM-18, is required for suppression of CO formation at the center region of the *C*. *elegans* chromosome (Saito and Colaiacovo 2014). We observed an increase in CO formation in the *slx4*∆ mutant as compared with wild type, specifically in those intervals that contain the centromere, on both chromosomes III and VII in budding yeast. In contrast to *C*. *elegans*, we did not observe this phenomenon in the *slx1*∆ mutant, nor in *rad1*∆ or *rtt107*∆. The Slx4-Slx1 complex plays a minor role in the resolution of Holliday junctions during a late step of meiotic recombination (Zakharyevich *et al.* 2012). In this study, we observed defects during an early step of meiosis in the *slx4*∆ mutant, such as the delayed formation of Rad51 foci (Figure 1E) and of meiotic DSB formation (Figure 4C), as well as a reduced amount of Spo11-oligo (Figure 1G). In addition, no elevation of Rad51 focus number at peak point in the *slx4*∆ mutant, even in the delayed recombination reaction (Figure 1F and Figure 4, D–H), also suggested reduced DSB formation or asynchronous DSB formation in the cells. In contrast, we did not observe any delay in the appearance of Zip1 foci (Figure 1D) and expression of Hop1 (Figure 2C). This indicates that the *slx4*∆ mutant has a defect in DSB formation but not in entry to meiosis. This result strongly suggests that Slx4 is involved in meiotic DSB formation and its regulation.

In meiotic DSB formation, the *slx4*∆ mutant showed an altered distribution of DSBs on chromosomes III and VII (Figure 5). Recently, it was reported that the Ctf19/CCAN subcomplex of the kinetochore protein complex is required to suppress centromere-proximal COs via DSB formation independently from the homologous chromosome pairing mediated by centromere-located Zip1 (Vincenten *et al.* 2015). In the case of *slx4*∆, we did not observe a clear correlation between the accumulation of meiotic DSBs and increase in CO formation. For example, we observed a high CO frequency in the *HIS4-MAT* interval, but we did not observe distinguishable differences in DSB distribution between *slx4*∆ and wild type. In addition, we did not observe any defect in the appearance of class I Zip1 (Figure S1A), which is the centromere-located form of Zip1, and is not dependent on the Ctf19/CCAN subcomplex (Vincenten *et al.* 2015). Thus, the functional relationship between Slx4 and the kinetochore complex in the suppression of centromere-proximal COs still remains unknown.

The finding that CO interference was abolished in the *Slx4*∆ cells, specifically in the intervals that contain the centromere in two different chromosomes (*HIS4-MAT* on chromosome III and *TRP5-ADE6* on chromosome VII), suggests that (i) abnormal CO formation was promoted in the centromere-proximal region in the absence of Slx4, or (ii) recruitment of the Msh4/Msh5 complex, which is an essential factor for CO control (Shinohara *et al.* 2008), to the DSBs might have been affected in this region. It is important to note that genetic NCO frequency was not affected in *slx4*∆ (Table S4). Thus, control-free CO formation would be activated in the centromere-proximal region in the absence of Slx4. In contrast, we observed stronger interference in the *URA3-LEU2* region not only in *slx4*∆, but also in *slx1*∆. COs in this interval originate from DSBs within a strong artificial *HIS4-LEU2* hot spot. This suggests that Slx1-Slx4 function might be involved in CO control, specifically at quite strong hot spots.

Slx4, with Rtt107 as a binding partner, functions as a negative regulator of Rad9 through competitive interaction with Dpb11 in the Mec1 pathway (Ohouo *et al.* 2013), and Slx4 phosphorylation is required for this function (Ohouo *et al.* 2010). However, Rad9 and Rad53 activities are excluded from the Spo11-dependent programmed DSB-related Mec1 activation pathway (Cartagena-Lirola *et al.* 2008). We observed an accumulation of Hop1 phosphorylation at T318, which is a Tel1/Mec1 phosphorylation site (Carballo and Cha 2007), in *slx4*∆, even with the slightly decreased amount of DSB formation at early time points. This suggested that Slx4-Rtt107 functions as a negative regulator of Mec1 activation even in the absence of Rad9 activation. Mec1 activation is required for negative regulation of Spo11-dependent meiotic DSB formation through Mec1 activation (Carballo *et al.* 2013). As we observed a slight reduction in the amount of Spo11-oligo in *slx4*∆, Rtt107-Slx4 might be involved in regulating the formation of meiotic DSBs.

Thus, Slx4 is required for the normal distribution of COs on each homolog-pair through meiotic DSB formation and CO control, especially in the centromere-proximal region.

## Supplementary Material

Supplemental Material
