# Supplementary material for: Budding Yeast SLX4 Contributes to the Appropriate Distribution of Crossovers and Meiotic Double-Strand Break Formation on Bivalents During Meiosis
Source: G3 (Bethesda). 2016 May 6;6(7):2033–42. doi: 10.1534/g3.116.029488 (PMC4938656; doi:10.1534/g3.116.029488)
Supplement: Supplemental Material [file supp_6_7_2033__index.html]

Budding Yeast SLX4 Contributes to the Appropriate Distribution of Crossovers and Meiotic Double-Strand Break Formation on Bivalents During Meiosis — Supplemental Material 

# Budding Yeast *SLX4* Contributes to the Appropriate Distribution of Crossovers and Meiotic Double-Strand Break Formation on Bivalents During Meiosis

## Supplemental Material for Higashide and Shinohara, 2016

**Files in this Data Supplement:**

- Figure S1 - Kinetics of appearance of Zip1 in each class. (.pdf, 651 KB)
- Figure S2 - FLAG-tagged SLX4 did not affect meiosis progression. (.pdf, 539 KB)
- Figure S3 - Meiotic DSB formation in the *slx4*Δ mutant. (.pdf, 516 KB)
- Table S1 - Strain list. (.pdf, 445 KB)
- Table S2 - Spore viability of *SLX4*-related mutants. (.pdf, 527 KB)
- Table S3 - Genetic analysis of *SLX4*-related mutant cells by Papazianï¿½s NPD analysis on chromosomes III and VII. (.pdf, 516 KB)
- Table S4 - Non-Mendelian segregation frequencies on chromosomes III and VII. (.pdf, 388 KB)
- Table S5 - Genetic analysis of *SLX4*-related mutant cells by coefficient of coincidence. (.pdf, 408 KB)
