# Supplementary material for: Budding Yeast SLX4 Contributes to the Appropriate Distribution of Crossovers and Meiotic Double-Strand Break Formation on Bivalents During Meiosis
Source: G3 (Bethesda). 2016 May 6;6(7):2033–42. doi: 10.1534/g3.116.029488 (PMC4938656; doi:10.1534/g3.116.029488)
Supplement: Supplemental Material [file supp_g3.116.029488_FigureS1.pdf]

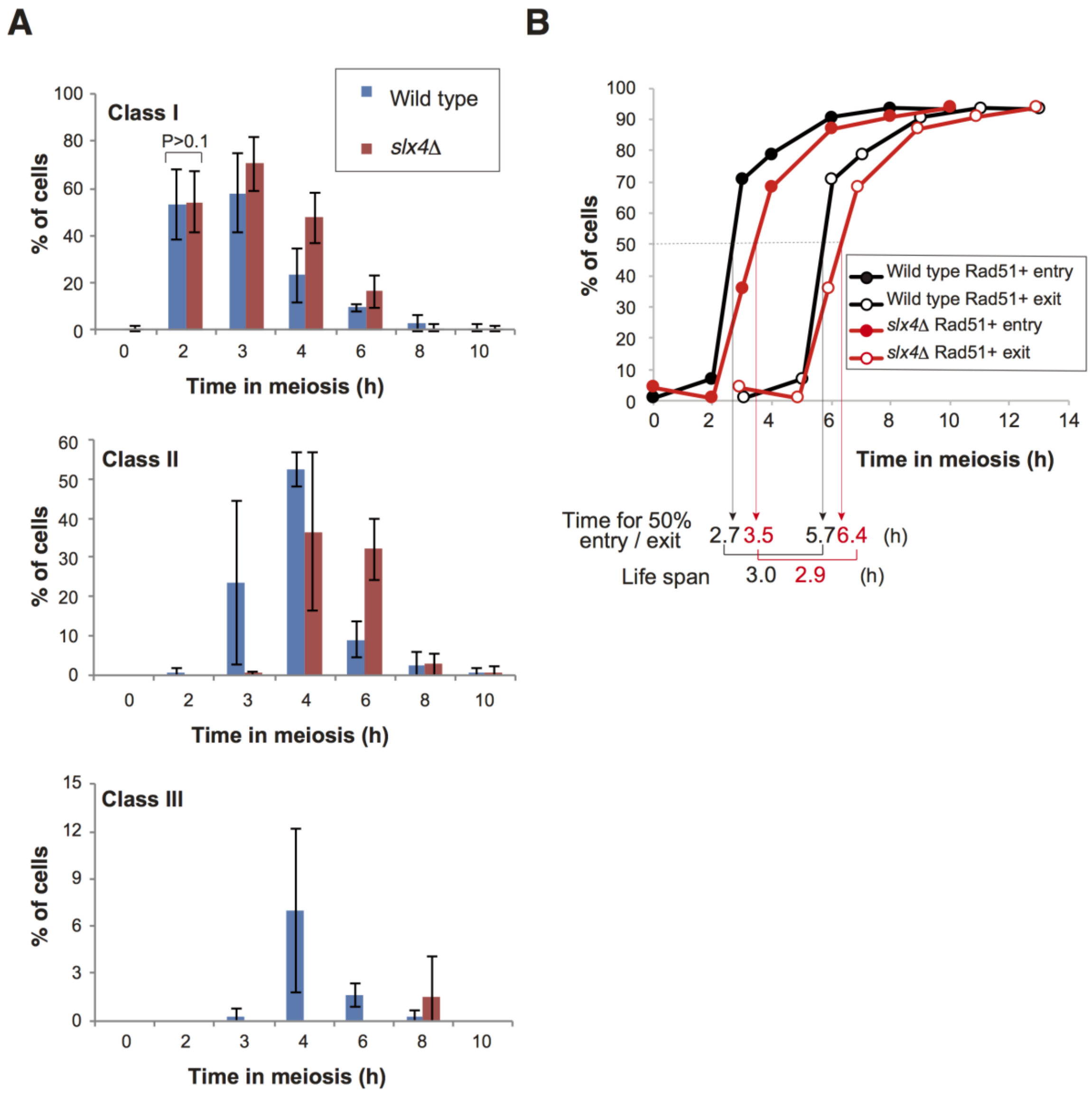
A
Wild type
slx4Δ
Class I
P>0.1
% of cells
Time in meiosis (h)
Class II
% of cells
Time in meiosis (h)
Class III
% of cells
Time in meiosis (h)
B
% of cells
Wild type Rad51+ entry
Wild type Rad51+ exit
slx4Δ Rad51+ entry
slx4Δ Rad51+ exit
Time in meiosis (h)
Time for 50% entry / exit
2.7 3.5 5.7 6.4 (h)
Life span 3.0 2.9 (h)

Figure S1 Kinetics of appearance of Zip1 in each class

(A) Averaged percentage of cells containing Zip1 in each class in indicated time points in wild type (NKY1551) and *slx4*Δ (MHY24) were shown. Error bars show the SD from three independent trials. Chi-square test was applied for statistical significance.

(B) Cumulative curve showed was converted from the non-cumulative curve in Figure 1E as described previously (SHINOHARA *et al.* 2000). Each time at which 50% of nuclei had entered or exited Rad51-positive stage was shown.
