# Supplementary material for: Budding Yeast SLX4 Contributes to the Appropriate Distribution of Crossovers and Meiotic Double-Strand Break Formation on Bivalents During Meiosis
Source: G3 (Bethesda). 2016 May 6;6(7):2033–42. doi: 10.1534/g3.116.029488 (PMC4938656; doi:10.1534/g3.116.029488)
Supplement: Supplemental Material [file supp_g3.116.029488_FigureS2.pdf]

Higashide M. et al Supplemental Figure-2

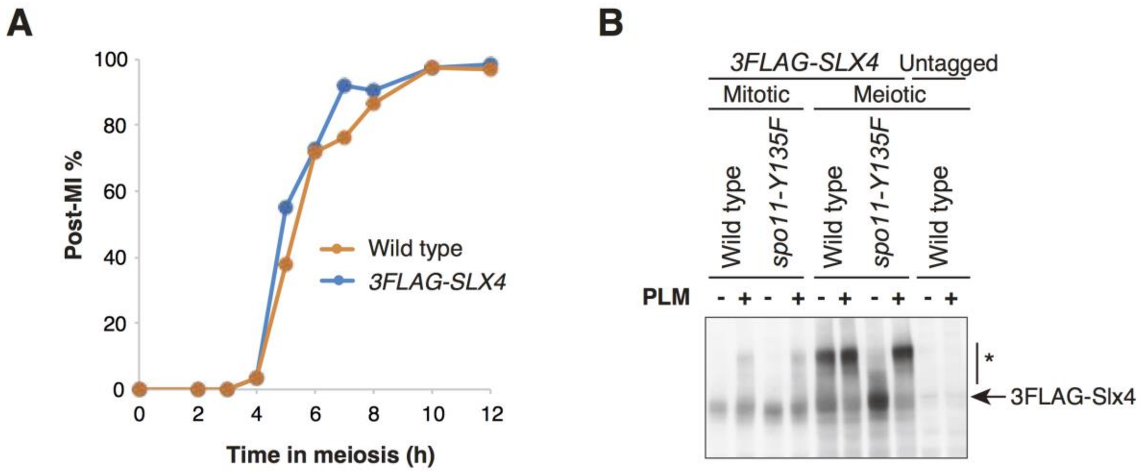

Figure S2 FLAG-tagged SLX4 did not affect meiosis progression.

(A) Representative meiosis progression is shown in wild type (NKY1551) and *3FLAG-SLX4* (MHY129), which was used for western blotting in Figure 2A.

(B) Phosphorylation of 3FLAG-Slx4 was analyzed in mitotic and meiotic cells. Whole-cell extracts from wild type (untagged; NKY1551), *3FLAG-SLX4* (MHY129), and *spo11-Y135F 3FLAG-SLX4* (MHY187) with or without phleomycin (PLM)-treatment, 20 μg/ml for mitotic cells or 5 μg/ml for meiotic cells (3.5 h), were analyzed as shown in Figure 2A.
