# Supplementary material for: Budding Yeast SLX4 Contributes to the Appropriate Distribution of Crossovers and Meiotic Double-Strand Break Formation on Bivalents During Meiosis
Source: G3 (Bethesda). 2016 May 6;6(7):2033–42. doi: 10.1534/g3.116.029488 (PMC4938656; doi:10.1534/g3.116.029488)
Supplement: Supplemental Material [file supp_g3.116.029488_FigureS3.pdf]

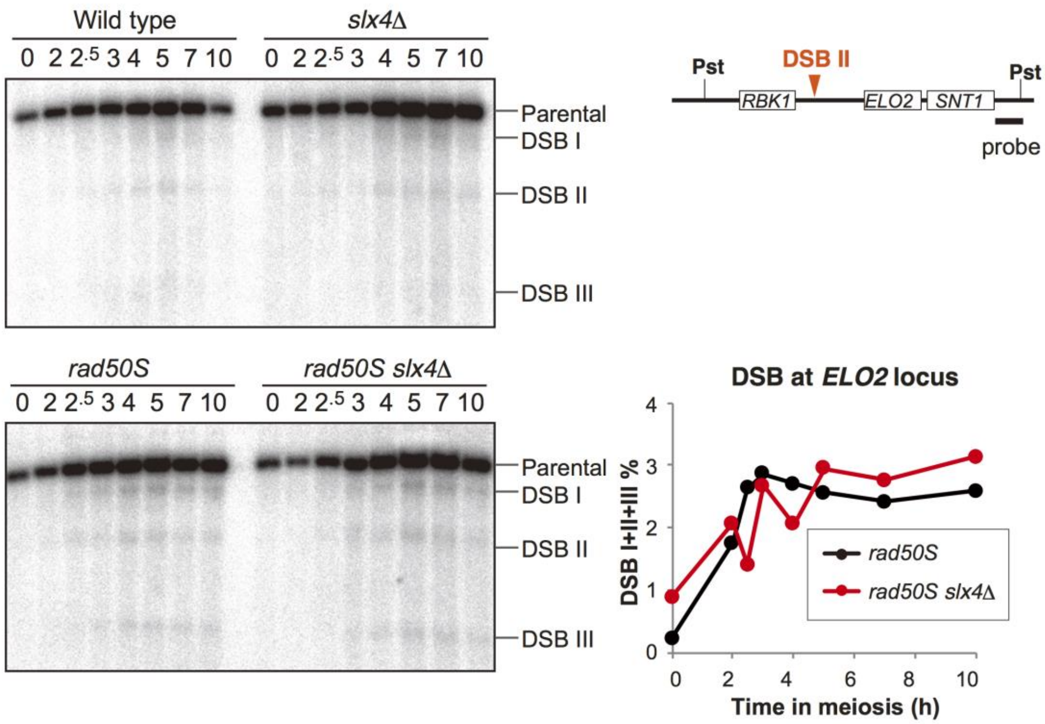
Wild type
slx4Δ
0 2 2.5 3 4 5 7 10
0 2 2.5 3 4 5 7 10
Parental
DSB I
DSB II
DSB III
Pst
DSB II
RBK1
ELO2
SNT1
Pst
probe
rad50S
rad50S slx4Δ
0 2 2.5 3 4 5 7 10
0 2 2.5 3 4 5 7 10
Parental
DSB I
DSB II
DSB III
DSB at ELO2 locus
DSB I+II+III %
rad50S
rad50S slx4Δ
Time in meiosis (h)
0 1 2 3 4
0 2 4 6 8 10

Figure S3 Meiotic DSB formation in the *slx*4Δ mutant.

(A) Representative Southern blotting of DSB detection at the *ELO2* locus in wild type (NKY1551) and *slx4*Δ (MHY24) (upper), or in *rad50S* (MSY1737) and *rad50S slx4*Δ (MHY77) (lower). Genetic map of the *ELO2* locus. Diagnostic restriction enzyme sites are shown as Pst (*Pst*I). Meiotic DSB I–III, as detected by the probe indicated the schematic, are identified for each blot. Sums of quantified relative amounts of DSBs at DSB I, II, and III in the *rad50S* background are shown in the graph.
