# Supplementary material for: Budding Yeast SLX4 Contributes to the Appropriate Distribution of Crossovers and Meiotic Double-Strand Break Formation on Bivalents During Meiosis
Source: G3 (Bethesda). 2016 May 6;6(7):2033–42. doi: 10.1534/g3.116.029488 (PMC4938656; doi:10.1534/g3.116.029488)
Supplement: Supplemental Material [file supp_g3.116.029488_TableS1.pdf]

Table S1 Strain list

Strain	Genotype	Reference
NKY1551	*MATa*/a, *ho::LYS2/", lys2/", ura3/", leu2::hisG/", his4X-LEU2(Bam*HI*)-URA3/his4B-LEU2(Mlu*I*), arg4-nsp/arg4-bgl*	(1)
MHY24	NKY1551 with *slx4::HYG*^{R}	This study
MHY68	NKY1551 with *slx1::TRP1*	This study
MHY82	NKY1551 with *mlh3::TRP1*	This study
MHY96	NKY1551 with *rad1::TRP1*	This study
MHY235	NKY1551 with *rtt107::TRP1*	This study
MHY365	NKY1551 with *spo11-Y135F-HA::KANMX, slx4::HYG*^{R}	This study
MHY129	NKY1551 with *3FLAG-SLX4*	This study
MHY187	NKY1551 with *3FLAG-SLX4, spo11-Y135F-HA::KANMX*	This study
MSY1737	NKY1551 with *rad50S::URA3*	(2)
MHY77	NKY1551 with *slx4::HYG*^{R}*, rad50S::URA3*	This study
MSY833/831	*MATa*/a, *ho::LYS2/", lys2/", ura3/", leu2::hisG/", trp1::hisG/"*	(3)
MSY5089	MSY833/831 with *SPO11-3FLAG::KANMX*	This study
MHY471	MSY833/831 with *SPO11-3FLAG::KANMX, slx4:: HYG*^{R}	This study
MSY4304	a, *ho::LYS2, lys2, his4B-leu2-E, cup2-B, met13-B, trp5-S, ade6-B, arg4-bgl*	This study
MSY4245	*a, ho::LYS2, lys2, hml::KanMX6, HIS4-LEU2-URA3, cyh2-R, arg4-bgl*	This study
MSY4930	MSY4304 with *slx4::HYG*^{R}	This study
MSY4910	MSY4245 with *slx4:: HYG*^{R}	This study
MSY5314	MSY4304 with *slx1:: HYG*^{R}	This study
MSY5282	MSY4245 with *slx1:: HYG*^{R}	This study
MSY5624	MSY4304 with *rad1::TRP1*	This study
MSY5622	MSY4245 with *rad1::TRP1*	This study
MSY5159	MSY4304 with *rtt107::TRP1*	This study
MSY5131	MSY4245 with *rtt107::TRP1*	This study

1. Storlazzi A, Xu L, Schwacha A, & Kleckner N (1996) Synaptonemal complex (SC) component Zip1 plays a role in meiotic recombination independent of SC polymerization along the chromosomes. *Proc Natl Acad Sci U S A* 93(17):9043-9048.
2. Shinohara M, Hayashihara K, Grubb JT, Bishop DK, & Shinohara A (2015) DNA damage response clamp 9-1-1 promotes assembly of ZMM proteins for formation of crossovers and synaptonemal complex. *J Cell Sci* 128(8):1494-1506.
3. Shinohara M, Sakai K, Ogawa T, & Shinohara A (2003) The mitotic DNA damage checkpoint proteins Rad17 and Rad24 are required for repair of double-strand breaks during meiosis in yeast. *Genetics* 164(3):855-865.
