# Supplementary material for: Budding Yeast SLX4 Contributes to the Appropriate Distribution of Crossovers and Meiotic Double-Strand Break Formation on Bivalents During Meiosis
Source: G3 (Bethesda). 2016 May 6;6(7):2033–42. doi: 10.1534/g3.116.029488 (PMC4938656; doi:10.1534/g3.116.029488)
Supplement: Supplemental Material [file supp_g3.116.029488_TableS2.pdf]

Table S2 Spore viability of *SLX4*-related mutants

| Strain | Tetrad type[a] 4-sv | 3-sv | 2-sv | 1-sv | 0-sv | Total# | Viability (%) | *P* value[b] |
|---|---|---|---|---|---|---|---|---|
| Wild type | 1134 | 163 | 39 | 2 | 3 | 1341 | 95.2 | |
| *slx4*Δ | 1414 | 203 | 40 | 3 | 22 | 1682 | 94.4 | 0.99 |
| *slx1*Δ | 1242 | 91 | 21 | 5 | 3 | 1362 | 97.1 | 0.99 |
| *Rad1*Δ | 1289 | 293 | 103 | 19 | 14 | 1724 | 90.8 | 0.98 |
| *rtt107*Δ | 1192 | 106 | 33 | 2 | 2 | 1192 | 96.5 | 0.99 |

[a] sv: spore viable in tetrad

[a]Statistical differences in the distribution of tetrad type as compared with that in wild type were calculated using the chi-square test.
