# Supplementary material for: Budding Yeast SLX4 Contributes to the Appropriate Distribution of Crossovers and Meiotic Double-Strand Break Formation on Bivalents During Meiosis
Source: G3 (Bethesda). 2016 May 6;6(7):2033–42. doi: 10.1534/g3.116.029488 (PMC4938656; doi:10.1534/g3.116.029488)
Supplement: Supplemental Material [file supp_g3.116.029488_TableS3.pdf]

Table S3 Genetic analysis of *SLX4*-related mutant cells by Papazian's NPD analysis on chromosomes III and VII

Chromosome III

| Strain (Total # of tetrads) | Interval | PD [a] | TT | $NPD_{obs}$ | Total | $NPD_{exp}$ | cM ± SEM [b] | NPD ratio ± SEM [b] |
|---|---|---|---|---|---|---|---|---|
| Wild type | *HML-URA3* | 878 | 242 | 1 | 1121 | 8 | 11.1 ± 0.7 | 0.13 ± 0.13 |
| (N=1134) | *URA3-LEU2* | 763 | 288 | 5 | 1056 | 12 | 15.1 ± 0.9 | 0.41 ± 0.18 |
| | *LEU2-HIS4* | 1021 | 34 | 0 | 1055 | 0 | 1.6 ± 0.3 | N.A. [a] |
| | *HIS4-MAT* | 434 | 645 | 30 | 1109 | 91 | 37.2 ± 1.5 | 0.33 ± 0.06 |
| | *HML-LEU2* | 573 | 477 | 12 | 1062 | 41 | 25.8 ± 1.2 | 0.29 ± 0.09 |
| | *URA3-HIS4* | 754 | 349 | 6 | 1109 | 18 | 17.4 ± 0.9 | 0.34 ± 0.14 |
| | *LEU2-MAT* | 402 | 622 | 31 | 1055 | 91 | 38.7 ± 1.6 | 0.34 ± 0.06 |
| | *HML-HIS4* | 566 | 536 | 15 | 1117 | 51 | 28.0 ± 1.2 | 0.29 ± 0.08 |
| *slx4*Δ | *HML-URA3* | 1291 | 100 | 1 | 1392 | 1 | 3.8 ± 0.4 | 1.06 ± 1.06 |
| (N=1414) | *URA3-LEU2* | 985 | 288 | 1 | 1274 | 10 | 11.5 ± 0.6 | 0.10 ± 0.10 |
| | *LEU2-HIS4* | 1229 | 51 | 0 | 1280 | 0 | 2.0 ± 0.3 | N.A. [a] |
| | *HIS4-MAT* | 312 | 959 | 108 | 1379 | 125 | 58.3 ± 2.0 | 0.86 ± 0.10 |
| | *HML-LEU2* | 915 | 371 | 4 | 1290 | 17 | 15.3 ± 0.8 | 0.24 ± 0.12 |
| | *URA3-HIS4* | 995 | 374 | 3 | 1372 | 16 | 14.3 ± 0.7 | 0.19 ± 0.11 |
| | *LEU2-MAT* | 272 | 902 | 105 | 1279 | 93 | 59.9 ± 2.1 | 1.13 ± 0.15 |

| | | | | | | | | |
|---|---|---|---|---|---|---|---|---|
| | *HML-HIS4* | 923 | 462 | 5 | 1390 | 25 | 17.7 ± 0.8 | 0.20 ± 0.09 |
| *slx1*Δ | *HML-URA3* | 1007 | 217 | 2 | 1226 | 5 | 9.3 ± 0.6 | 0.36 ± 0.26 |
| (N=1032) | *URA3-LEU2* | 808 | 330 | 6 | 1144 | 15 | 16.0 ± 0.9 | 0.40 ± 0.16 |
| | *LEU2-HIS4* | 1090 | 55 | 0 | 1145 | 0 | 2.4 ± 0.3 | N.A. [a] |
| | *HIS4-MAT* | 470 | 697 | 50 | 1217 | 95 | 41.0 ± 1.7 | 0.53 ± 0.08 |
| | *HML-LEU2* | 641 | 493 | 18 | 1152 | 39 | 26.1 ± 1.3 | 0.46 ± 0.11 |
| | *URA3-HIS4* | 798 | 407 | 9 | 1214 | 23 | 19.0 ± 0.1 | 0.40 ± 0.13 |
| | *LEU2-MAT* | 409 | 685 | 50 | 1144 | 105 | 43.1 ± 1.7 | 0.48 ± 0.07 |
| | *HML-HIS4* | 633 | 570 | 23 | 1226 | 52 | 28.9 ± 1.3 | 0.44 ± 0.10 |
| *rad1*Δ | *HML-URA3* | 994 | 269 | 4 | 1267 | 8 | 11.6 ± 0.7 | 0.48 ± 0.24 |
| (N=1032) | *URA3-LEU2* | 846 | 346 | 2 | 1194 | 16 | 15.0 ± 0.7 | 0.13 ± 0.09 |
| | *LEU2-HIS4* | 1150 | 54 | 0 | 1204 | 0 | 2.2 ± 0.3 | N.A. [a] |
| | *HIS4-MAT* | 504 | 722 | 40 | 1266 | 98 | 38.0 ± 1.5 | 0.41 ± 0.07 |
| | *HML-LEU2* | 634 | 565 | 14 | 1213 | 51 | 26.8 ± 1.1 | 0.27 ± 0.07 |
| | *URA3-HIS4* | 832 | 421 | 4 | 1257 | 23 | 17.7 ± 0.8 | 0.17 ± 0.09 |
| | *LEU2-MAT* | 437 | 716 | 49 | 1202 | 108 | 42.0 ± 1.7 | 0.45 ± 0.07 |
| | *HML-HIS4* | 622 | 638 | 17 | 1277 | 66 | 29.0 ± 1.1 | 0.26 ± 0.06 |
| *rtt107*Δ | *HML-URA3* | 942 | 221 | 1 | 1164 | 6 | 9.7 ± 0.6 | 0.17 ± 0.17 |
| (N=1192) | *URA3-LEU2* | 800 | 290 | 4 | 1094 | 12 | 14.4 ± 0.8 | 0.34 ± 0.17 |
| | *LEU2-HIS4* | 1047 | 57 | 0 | 1104 | 0 | 2.6 ± 0.3 | N.A. [a] |

…

| | | | | | | | | |
|---|---|---|---|---|---|---|---|---|
| | *HIS4-MAT* | 482 | 648 | 33 | 1163 | 83 | 36.4 ± 1.5 | 0.40 ± 0.07 |
| | *HML-LEU2* | 628 | 469 | 10 | 1107 | 37 | 23.9 ± 1.1 | 0.27 ± 0.09 |
| | *URA3-HIS4* | 776 | 378 | 4 | 1158 | 20 | 17.4 ± 0.8 | 0.20 ± 0.10 |
| | *LEU2-MAT* | 418 | 645 | 39 | 1102 | 93 | 39.9 ± 1.7 | 0.42 ± 0.07 |
| | *HML-HIS4* | 607 | 551 | 13 | 1171 | 51 | 26.9 ± 1.1 | 0.25 ± 0.07 |

Chromosome VII

| Strain (Total # of tetrad) | Intervals | PD | TT | $NPD_{obs}$ | Total | $NPD_{exp}$ | cM ± SEM [b] | NPD ratio ± SEM [b] |
|---|---|---|---|---|---|---|---|---|
| Wild type (N=1134) | *CUP2-MET13* | 440 | 564 | 18 | 1022 | 71 | 32.9 ± 1.4 | 0.25 ± 0.06 |
| | *MET13-CYH2* | 822 | 250 | 1 | 1073 | 9 | 11.9 ± 0.7 | 0.11 ± 0.11 |
| | *CYH2-TRP5* | 377 | 695 | 27 | 1099 | 126 | 39.0 ± 1.4 | 0.21 ± 0.05 |
| | *TRP5-ADE6* | 330 | 694 | 64 | 1092 | 132 | 49.5 ± 2.0 | 0.49 ± 0.08 |
| | *CUP12-CYH2* | 324 | 694 | 40 | 1058 | 148 | 44.1 ± 1.7 | 0.27 ± 0.05 |
| *slx4*Δ (N=1414) | *CUP2-MET13* | 691 | 556 | 20 | 1267 | 46 | 26.7 ± 1.2 | 0.44 ± 0.10 |
| | *MET13-CYH2* | 1005 | 338 | 6 | 1349 | 13 | 13.9 ± 0.8 | 0.47 ± 0.19 |
| | *CYH2-TRP5* | 445 | 892 | 50 | 1387 | 173 | 43.0 ± 1.5 | 0.29 ± 0.05 |
| | *TRP5-ADE6* | 234 | 957 | 180 | 1371 | 118 | 74.3 ± 2.4 | 1.52 ± 0.20 |
| | *CUP12-CYH2* | 482 | 775 | 47 | 1304 | 116 | 40.5 ± 1.5 | 0.40 ± 0.07 |

| | | | | | | | | |
|---|---|---|---|---|---|---|---|---|
| *slx1*Δ | *CUP2-MET13* | 505 | 578 | 18 | 1101 | 65 | 31.2 ± 1.3 | 0.28 ± 0.07 |
| (N=1032) | *MET13-CYH2* | 931 | 241 | 1 | 1173 | 7 | 10.5 ± 0.6 | 0.14 ± 0.14 |
| | *CYH2-TRP5* | 421 | 766 | 29 | 1216 | 137 | 38.7 ± 1.4 | 0.21 ± 0.04 |
| | *TRP5-ADE6* | 321 | 781 | 89 | 1191 | 167 | 55.1 ± 2.2 | 0.53 ± 0.08 |
| | *CUP12-CYH2* | 402 | 710 | 34 | 1146 | 120 | 33.9 ± 1.5 | 0.28 ± 0.05 |
| *rad1*Δ | *CUP2-MET13* | 533 | 631 | 12 | 1176 | 75 | 29.9 ± 1.1 | 0.16 ± 0.05 |
| (N=1032) | *MET13-CYH2* | 945 | 295 | 0 | 1240 | 11 | 11.9 ± 0.6 | <0.09 |
| | *CYH2-TRP5* | 459 | 765 | 43 | 1267 | 120 | 40.4 ± 1.5 | 0.36 ± 0.06 |
| | *TRP5-ADE6* | 352 | 830 | 67 | 1249 | 196 | 49.3 ± 1.8 | 0.34 ± 0.06 |
| | *CUP12-CYH2* | 385 | 789 | 33 | 1207 | 165 | 40.9 ± 1.4 | 0.20 ± 0.04 |
| *rtt107*Δ | *CUP2-MET13* | 465 | 581 | 10 | 1056 | 72 | 30.4 ± 1.1 | 0.14 ± 0.04 |
| (N=1192) | *MET13-CYH2* | 845 | 272 | 3 | 1120 | 10 | 12.9 ± 0.8 | 0.30 ± 0.17 |
| | *CYH2-TRP5* | 392 | 731 | 33 | 1156 | 133 | 40.2 ± 1.5 | 0.25 ± 0.05 |
| | *TRP5-ADE6* | 311 | 731 | 85 | 1127 | 147 | 55.1 ± 2.2 | 0.58 ± 0.08 |
| | *CUP12-CYH2* | 337 | 725 | 37 | 1099 | 161 | 43.1 ± 1.6 | 0.23 ± 0.05 |

Map distances and NPD ratios were calculated as described in the Material and Methods. NPDobs is the number of NPDs observed in crosses. NPDexp is the number of NPDs expected from Papazian's equation.

[a] PD: parental di-type, TT:tetra type, NPD: non-parental di-type, N.A.: not applicable

[b] The SEM for the map distances and NPD ratio were calculated using the Stahl Lab online tool (http://www.molbio.uoregon.edu/~fstahl).
