# Supplementary material for: Budding Yeast SLX4 Contributes to the Appropriate Distribution of Crossovers and Meiotic Double-Strand Break Formation on Bivalents During Meiosis
Source: G3 (Bethesda). 2016 May 6;6(7):2033–42. doi: 10.1534/g3.116.029488 (PMC4938656; doi:10.1534/g3.116.029488)
Supplement: Supplemental Material [file supp_g3.116.029488_TableS4.pdf]

Table S4 Non-Mendelian segregation frequencies on chromosomes III and VII

Strain	Chromosome III						Chromosome VII				Total# of tetrad
	HML	*URA3*	*LEU2*	*HIS4*	*MAT*	*CUP2*	*MET13*	*CYH2*	*TRP5*	*ADE6*	
Wild type	3 (1.0)	11 (1.0)	69 (1.0)	14 (1.0)	11 (1.0)	66 (1.0)	51 (1.0)	14 (1.0)	23 (1.0)	23 (1.0)	1134
*slx4*Δ	2 (0.3)	20 (1.6)	122 (1.4)	22 (1.3)	13 (0.9)	96 (1.2)	52 (0.8)	13 (0.8)	14 (0.5)	29 (1.1)	1414
*slx1*Δ	2 (1.0)	14 (1.4)	88 (1.2)	14 (1.1)	11 (0.8)	87 (1.2)	59 (1.1)	10 (0.9)	17 (0.8)	34 (1.6)	1242
*rad1*Δ	2 (0.7)	22 (1.9)	76 (1.0)	12 (0.8)	14 (1.1)	76 (1.0)	41 (0.7)	10 (0.7)	14 (0.6)	29 (1.1)	1291
*rtt107*Δ	9 (2.6)	22 (2.0)	77 (1.1)	13 (0.9)	16 (1.3)	81 (1.2)	61 (1.1)	13 (0.9)	25 (1.1)	41 (1.7)	1192

Numbers of tetrad-type with 3+:1–, 1+:3–, 4+:0–, and 0+:4– segregation for each marker were shown. The ratios of the frequency in the mutant relative to that in the wild type are shown in parentheses.
