# Supplementary material for: Budding Yeast SLX4 Contributes to the Appropriate Distribution of Crossovers and Meiotic Double-Strand Break Formation on Bivalents During Meiosis
Source: G3 (Bethesda). 2016 May 6;6(7):2033–42. doi: 10.1534/g3.116.029488 (PMC4938656; doi:10.1534/g3.116.029488)
Supplement: Supplemental Material [file supp_g3.116.029488_TableS5.pdf]

Table S5 Genetic analysis of *SLX4*-related mutant cells by the coefficient of coincidence

Chromosome III

	Frequency of consecutive COs observed			Frequency of consecutive COs expected[a]			Ratio of observed/expected		
Intervals	*HML-URA3-LEU2*	*URA3-LEU2-HIS4*	*LEU2-HIS4-MAT*	*HML-URA3-LEU2*	*URA3-LEU2-HIS4*	*LEU2-HIS4-MAT*	*HML-URA3-LEU2*	*URA3-LEU2-HIS4*	*LEU2-HIS4-MAT*
Wild type	0.061	0.009	0.020	0.030	0.005	0.015	0.491	0.535	0.733
*slx4*Δ	0.018	0.009	0.030	0.013	0.007	0.023	0.724	0.806	0.773
*slx1*Δ	0.054	0.014	0.030	0.026	0.011	0.020	0.475	0.754	0.688
*rad1*Δ	0.065	0.013	0.027	0.032	0.010	0.015	0.485	0.781	0.560
*rtt107*Δ	0.053	0.014	0.032	0.026	0.011	0.018	0.488	0.791	0.558

Chromosome VII

	Frequency of consecutive COs observed			Frequency of consecutive COs expected[a]			Ratio of observed/expected		
Intervals	*CUP2-MET13-CYH2*	*MET13-CYH2-TRP5*	*CYH2-TRP5-ADE6*	*CUP2-MET13-CYH2*	*MET13-CYH2-TRP5*	*CYH2-TRP5-ADE6*	*CUP2-MET13-CYH2*	*MET13-CYH2-TRP5*	*CYH2-TRP5-ADE6*

Wild type	0.130	0.145	0.444	0.085	0.123	0.434	0.655	0.846	0.977
*slx4*Δ	0.114	0.166	0.549	0.068	0.133	0.544	0.598	0.799	0.991
*slx1*Δ	0.112	0.136	0.477	0.078	0.117	0.465	0.701	0.858	0.976
*rad1*Δ	0.128	0.149	0.453	0.081	0.127	0.432	0.636	0.854	0.954
*rtt107*Δ	0.138	0.162	0.475	0.097	0.128	0.461	0.702	0.791	0.970

[a] Expected frequencies of consecutive COs were calculated by multiplication of the individual CO frequencies from the two consecutive intervals as indicated. Statistical significance was calculated by *G*-test. *$P<0.05$
